# Obesity and Long COVID: intersecting epidemics?

**DOI:** 10.1186/s12889-025-26134-1

**Published:** 2026-01-22

**Authors:** Michael Gottlieb, Huihui Yu, Ji Chen, Erica S. Spatz, Nicole L. Gentile, Rachel E. Geyer, Michelle Santangelo, Caitlin Malicki, Kristyn Gatling, Kelli N. O’Laughlin, Kari A. Stephens, Joann G. Elmore, Lauren E. Wisk, Michelle L’Hommedieu, Robert M. Rodriguez, Juan Carlos C. Montoy, Ralph C. Wang, Kristin L. Rising, Efrat Kean, Jonathan W. Dyal, Mandy J. Hill, Arjun K. Venkatesh, Robert A. Weinstein, INSPIRE Group

**Affiliations:** 1 Department of Emergency Medicine, Rush University Medical Center, 1750 West Harrison Street, Suite 108 Kellogg, Chicago, IL 60612 USA; 2 Section of Cardiovascular Medicine, Yale School of Medicine New Haven, New Haven, CT USA; 3 Yale Center for Outcomes Research and Evaluation, New Haven, CT USA; 4 Department of Family Medicine, University of Washington, Seattle, WA USA; 5 Department of Laboratory Medicine and Pathology, University of Washington, Seattle, WA USA; 6 Department of Emergency Medicine, Yale School of Medicine, New Haven, CT USA; 7 Division of Infectious Diseases, Department of Medicine, Rush University Medical Center, Chicago, IL USA; 8 Departments of Emergency Medicine and Global Health, University of Washington, Seattle, WA USA; 9 Division of General Internal Medicine and Health Services Research, David Geffen School of Medicine at UCLA, Los Angeles, CA USA; 10 Department of Internal Medicine, University of California, Riverside, Riverside, CA USA; 11 Department of Emergency Medicine, University of California, San Francisco, San Francisco, CA USA; 12 Department of Emergency Medicine, Sidney Kimmel Medical College, Philadelphia, PA USA; 13 Center for Connected Care, Thomas Jefferson University, Philadelphia, PA USA; 14 Department of Emergency Medicine, Johns Hopkins University School of Medicine, Baltimore, MD USA; 15 School of Public and Population Health, University of Texas Medical Branch at Galveston, Galveston, TX USA; 16 Department of Medicine, Cook County Hospital, Chicago, IL USA

**Keywords:** Long COVID, COVID-19, SARS-CoV-2, Obesity

## Abstract

**Background:**

Obesity affects over 10% of the world population and has significant public health implications. With rising recognition of the long-term effects of Long COVID (LC) coupled with new agents to facilitate weight loss, it is critical to understand the influence of obesity on LC. This study assessed the association of obesity with rates of LC and degree of LC-related mental and physical health outcomes among participants up to three years after initial infection.

**Methods:**

This was a cross-sectional, multisite study of participants with SARS-CoV-2 infection from 12/11/2020–8/29/2022, with data collected through 4/2/2024. Surveys included validated tools for physical and mental health. Data were analyzed by self-reported new obesity (follow-up only), persistent obesity (baseline and follow-up), or no obesity.

**Results:**

Of 3,663 participants, 547 (14.9%) had new obesity and 805 (21.9%) had persistent obesity. Compared with persons without obesity, LC was significantly more common among those with new (39.7% vs 22.8%; aOR: 1.9, 95% CI 1.5–2.4) or persistent obesity (39.1% vs 22.8%; aOR: 1.7, 95% CI 1.4–2.1). Regardless of chronicity and current LC status, obesity was associated with lower (worse) scores for PROMIS Physical (mean differences: 2.7–4.0) and Mental Health (mean differences: 1.7–3.6) function, worse moderate-to-severe fatigue (aOR: 1.3–2.1), worse dyspnea (aOR: 1.9–3.7), worse loneliness (aOR: 1.3–1.6), and insufficient activity (aOR for SNAP ≤ 4: 1.6–2.8; aOR for EVS ≤ 150 min/week: 2.0–3.1).

**Conclusions:**

Participants with obesity had higher rates of LC and worse physical and mental health outcomes, regardless of LC status. These findings raise key questions about obesity interventions to treat LC and a possible role for obesity management before the next pandemic.

**Trial registration:**

NCT04610515

**Supplementary Information:**

The online version contains supplementary material available at 10.1186/s12889-025-26134-1.

## Background

As of May 2025, there have been over 777 million reported cases of COVID-19 worldwide [1]. Approximately 1-in-10 people with severe acute respiratory syndrome coronavirus-2 (SARS-CoV-2) will develop symptoms lasting at least three months post-infection, which can have a substantial impact on their quality of life, a condition commonly referred to as Long COVID (LC) [2–10].

At the same time, obesity is an increasing public health issue. Currently, 1-in-8 persons worldwide are living with obesity, a frequency that has tripled since 1990, [11, 12] and over 40% of United States citizens are experiencing obesity [13]. Obesity is already a recognized risk factor for more severe acute SARS-CoV-2 infections [14–17]. The extent to which obesity influences the incidence and severity of LC is not known. As recent pharmacologic interventions have emerged to assist with weight loss, [18] understanding the association between obesity and LC to guide risk stratification and focused interventions among patients with obesity is needed.

To address these gaps, we utilized data from the Innovative Support for Patients with SARS-CoV-2 Infections Registry (INSPIRE) to analyze the associations between obesity and the rates and severity of LC.

## Methods

### Study design

INSPIRE was a prospective, longitudinal study conducted across eight major healthcare institutions in the United States that were selected for diversity of geographic location and participant populations (Appendix). Participants were aged  ≥ 18 years with SARS-CoV-2 infection between 12/7/2020–8/29/2022. Details of participant recruitment, study methods, and surveys have been previously published (https://www.clinicaltrials.gov; NCT04610515; Registered: 10/28/2020) [19]. Long-term follow-up surveys were completed 2/27/2024–4/2/2024, which was 18–40 months after index infection. INSPIRE was funded by the Centers for Disease Control and Prevention (CDC) and received institutional review board approval at all eight institutions. All participants provided electronic written informed consent to participate. The study adhered to the Strengthening the Reporting of Observational Studies in Epidemiology (STROBE) guidelines [20].

### Study outcomes

All data were collected in the long-term survey, except for demographics (e.g., age, gender, race, ethnicity), which were collected on the baseline survey at initial study enrollment. LC status was determined by the following question: “Following COVID-19 infections, some people may develop a condition called Long COVID. This is defined as having symptoms (such as fatigue, shortness of breath, brain fog, etc.) that last for more than 12 weeks or having symptoms that suddenly emerge without another explanation. This condition is called Long COVID. Do you think you have had Long COVID?” (yes or no). We intentionally utilized self-report of LC to be consistent with more recent definitions, which emphasize the multitude of potential symptoms and important role of patient involvement in defining LC [10]. Current LC was defined as responding “yes” to the above item. Those responding “no” (regardless of prior history of resolved LC) were considered not to have current LC. Obesity also was based upon self-report from patients (defined as the presence or absence) using survey data at baseline and follow-up (18–40 months). “No obesity” was defined as not having obesity at baseline or follow-up (inclusive of resolved obesity). “New obesity” was defined as obesity that was not present on the baseline survey and only present at the long-term follow-up survey, whereas “persistent obesity” was defined as occurring with both surveys.

The survey tools were developed by study investigators, informed by the existing literature and employed validated tools. A patient advisory board reviewed the survey items and provided focused feedback to establish content and response process validity. We assessed eight patient-reported outcome measures as indicators of participant physical and mental health status.

For physical health, we collected: Patient-Reported Outcomes Measurement Information System (PROMIS)−29 version 2.1 Physical Health global score [21], Fatigue Severity Scale (FSS) [22], and the Modified Medical Research Council (MMRC) Dyspnea scale [23]. For mental health status, we used the PROMIS-29 version 2.1 Mental Health global score. [21]

The PROMIS-29 tool uses a T-score metric, where 50 represents the mean score of a reference population with a standard deviation of 10 [24, 25]. For PROMIS-29 Physical Health and Mental Health scoring, higher scores correspond to lesser severity (i.e., higher scores are better). Based upon existing literature, we considered a clinically-important difference in PROMIS-29 scores to be ≥ 2 T-score points [26].

Fatigue Severity Scale is a 9-item tool, with each item being scored of 1–7 (total score: 9–63), where none/mild fatigue is ≤ 35, moderate fatigue is 36–52, and severe fatigue is ≥ 53 [27]. A clinically significant FSS score is defined as ≥ 36 [27].

The MMRC Dyspnea scale is a single-item tool that asks participants to select when they become short of breath, ranging from 0 (“I only get breathless with strenuous exercise”) to 4 (“I am too breathless to leave the house” or “I am breathless when dressing”). Higher MMRC Dyspnea scores suggesting more severe impairment from dyspnea.

### Statistical analysis

We examined differences in participant demographics, LC status, and patient-reported mental and physical health outcomes across non-obese, new obesity, and persistent obesity groups. Categorical variables were compared using chi-square tests, while continuous variables were assessed using Kruskal–Wallis tests.

To assess the association between obesity and the presence of LC, we performed logistic regression adjusting for age, gender, race/ethnicity, SARS-CoV-2 variant at the time of initial infection, vaccination status before infection, and total number of vaccine doses received prior to their first infection. Adjusted odds ratios (aORs) with 95% confidence intervals (CIs) were reported to quantify the association between obesity status and LC.

We further explored the association of obesity with mental and physical health outcomes. Linear regression models were used for continuous outcomes, reporting adjusted least-square (LS) mean differences across obesity groups. For binary outcomes, logistic regression models were used to report adjusted odds ratios. In addition to the covariates included in the LC model, an interaction term between obesity status and LC status was incorporated to evaluate whether the effect of obesity differed by LC status.

All statistical analyses were conducted using SAS version 9.4 (SAS Institute Inc., Cary, NC) and R version 4.3.3. Visualizations were created using Microsoft Excel. Given the exploratory nature of the study, no adjustments were made for multiple comparisons. All tests were two-sided, with a significance threshold of *p* < 0.05.

### Role of the funding source

The funder (Centers for Disease Control and Prevention) assisted with the design and conduct of the study. The funder had no role regarding the collection, management, analysis, and interpretation of the data; preparation, review, or approval of the manuscript; and decision to submit the manuscript for publication.

## Results

**Fig. 1:**
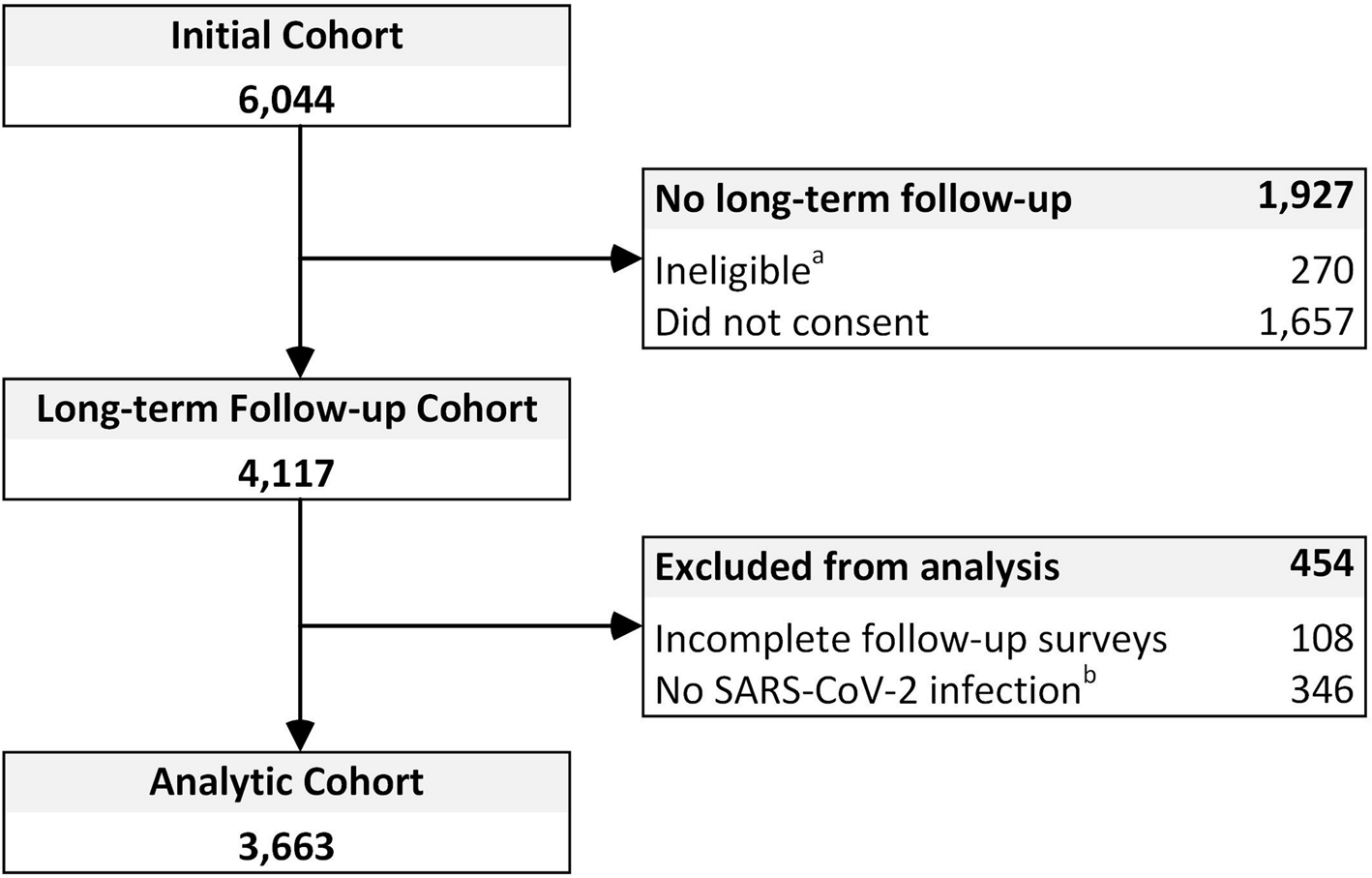
Flow diagram of enrollment. **a** Ineligible to receive consent addendum invitation due to being withdrawn or deceased at the end of the original study phase, opted out of study extension communications or did not opt in to receive the invitation (University of Texas Southwestern site only); **b** No SARS-CoV-2 infection reported on the long-term survey

Among 4117 INSPIRE participants who consented to receive the long-term survey, 3663 (91%) reported at least one SARS-CoV-2 infection since enrollment and were eligible for this analysis (Fig. 1). The mean age at initial enrollment was 40 years and 66% of participants were female. Overall, 66.6% were White, 13.9% were Hispanic/Latino, 13.6% were Asian, 7.7% were Black or African American, and 9.1% self-identified as another or multiple races.

In total, 2,311 (63.1%) had no obesity, 805 (21.9%) had persistent obesity, and 547 (14.9%) had new obesity reported at long term follow-up. In general, the cohort without obesity was slightly younger, less likely to be Hispanic/Latino, and more likely to have been infected with the Omicron variant. Full demographics by obesity status are included in Table 1.

**Table 1:** Demographic characteristics by self-reported obesity status

Demographics	Category	No Obesity (*N* = 2,311)	New Obesity (*N* = 547)	Persistent Obesity (*N* = 805)	*p-value*
Age	Mean (SD)	38.5 (14.3)	41.9 (13.8)	43.8 (13.7)	< 0.001
	18 to 34	1121 (48.5%)	190 (34.7%)	234 (29.1%)	< 0.001
	35 to 49	676 (29.3%)	197 (36.0%)	307 (38.1%)	
	50 to 64	355 (15.4%)	116 (21.2%)	191 (23.7%)	
	65 +	159 (6.9%)	44 (8.0%)	73 (9.1%)	
Gender	Female	1493 (64.6%)	358 (65.4%)	578 (71.8%)	< 0.001
	Male	718 (31.1%)	167 (30.5%)	182 (22.6%)	
	Transgender/Non-binary/Other	100 (4.3%)	22 (4.0%)	45 (5.6%)	
Race/Ethnicity	White	1353 (58.5%)	271 (49.5%)	490 (60.9%)	< 0.001
	Black or African American	113 (4.9%)	73 (13.3%)	75 (9.3%)	
	Asian	398 (17.2%)	42 (7.7%)	42 (5.2%)	
	Other/Multiple	122 (5.3%)	31 (5.7%)	48 (6.0%)	
	Hispanic/Latino	269 (11.6%)	109 (19.9%)	132 (16.4%)	
	Not Reported	56 (2.4%)	21 (3.8%)	18 (2.2%)	
Variant	Pre-Delta	257 (11.1%)	107 (19.6%)	120 (14.9%)	< 0.001
	Delta	773 (33.4%)	165 (30.2%)	285 (35.4%)	
	Omicron	1281 (55.4%)	275 (50.3%)	400 (49.7%)	
Vaccinated before initial SARS-CoV-2 infection		1412 (80.1%)	310 (74.2%)	509 (77.4%)	0.02
Total Doses of SARS-CoV-2 Vaccination	Mean (SD)	3.7 (1.5)	3.5 (1.7)	3.6 (1.6)	0.0151

*SD* standard deviation

**Fig. 2:**
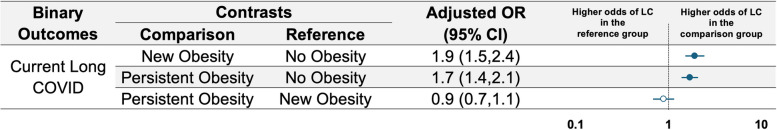
Adjusted odds ratio for the presence of Long COVID among participants with new obesity, persistent obesity, or no obesity. OR, odds ratio; CI, confidence interval; LC, Long COVID; Adjustment included age, gender, race/ethnicity, variant, SARS-CoV-2 vaccination status before the initial infection, and number of vaccination doses

Current LC was present in 527 participants (22.8%) without obesity, 315 (39.1%) with persistent obesity, and 217 (39.7%) with new obesity (Table 2). When compared to participants without obesity, those with new obesity (aOR: 1.9; 95% CI 1.5 to 2.4) and persistent obesity (aOR: 1.7; 95% CI 1.4 to 2.1) were more likely to have current LC (Fig. 2; eFigure 1).

**Table 2:** Outcome data by self-reported obesity status at follow-up

Outcome	No Obesity (*N* = 2,311)	New Obesity (*N* = 547)	Persistent Obesity (*N* = 805)	*p-value*
**Continuous Outcome; mean (SD)**				
***Higher scores are better***				
PROMIS Physical Health Global Score	53.6 (6.9)	48.6 (8.7)	48.3 (8.9)	< 0.001
PROMIS Mental Health Global Score	53.0 (8.8)	48.3 (9.8)	48.1 (9.8)	< 0.001
***Lower scores are better***				
Fatigue severity scale scores	24.4 (17.0)	33.1 (18.1)	33.1 (18.1)	< 0.001
**Dichotomous Outcome; n (%)**				
Current Long COVID	527 (22.8%)	217 (39.7%)	315 (39.1%)	< 0.001
Fatigue Severity Scale ≥ 36	632 (27.3%)	255 (46.6%)	381 (47.3%)	< 0.001
**Categorical Outcome; n (%)**				
MMRC Dyspnea Scale				< 0.001
[1]* I only get breathless with strenuous exercise*	1580 (68.4%)	237 (43.3%)	288 (35.8%)	
[2]* I get short of breath when hurrying or walking up a slight hill*	641 (27.7%)	235 (43.0%)	388 (48.2%)	
[3]* I walk slower than people of the same age because of breathlessness or have to stop for breath when walking at my own pace*	66 (2.9%)	51 (9.3%)	97 (12.0%)	
[4]* I stop for breath after walking about 100 yards or after a few minutes*	16 (0.7%)	19 (3.5%)	27 (3.4%)	
[5]* I am too breathless to leave the house" or "I am breathless when dressing"*	8 (0.3%)	5 (0.9%)	5 (0.6%)	

**Fig. 3:**
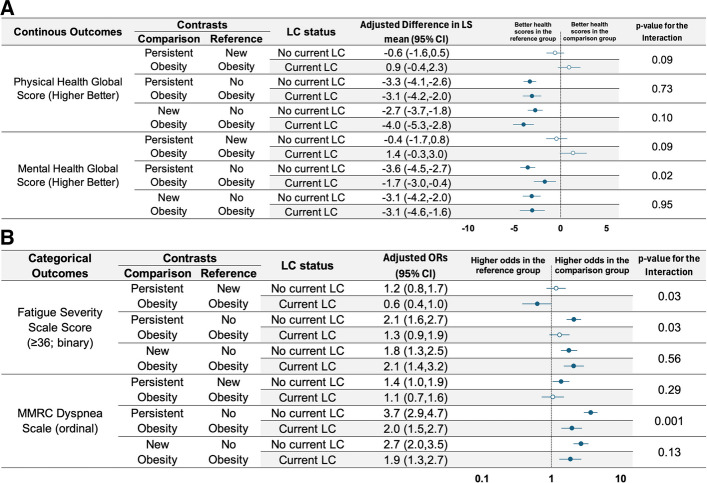
**A** Adjusted least-squares mean differences in PROMIS physical and mental health scores among participants with new obesity, persistent obesity, or no obesity. LS, least squares; CI, confidence interval; LC, Long COVID; Adjustment included age, gender, race/ethnicity, variant, SARS-CoV-2 vaccination status before the initial infection, and number of vaccination doses; The p-value is used to test the significance of the interaction between obesity status and LC status (i.e., to assess whether the contrast between obesity groups differs significantly between the No Current LC group and Current LC group). **B **Adjusted odds ratio for severe fatigue and dyspnea among participants with new obesity, persistent obesity, or no obesity. OR, odds ratio; CI, confidence interval; LC, Long COVID; Adjustment included age, gender, race/ethnicity, variant, SARS-CoV-2 vaccination status before the initial infection, and number of vaccination doses; The p-value is used to test the significance of the interaction between obesity status and LC status (i.e., to assess whether the contrast between obesity groups differs significantly between the No Current LC group and Current LC group)

Compared to those without obesity, the PROMIS physical health score in the current LC cohort was worse in the new obesity group (−4.0 points; 95% CI −5.3 to −2.8) and in the persistent obesity group (−3.1 points; 95% CI −4.2 to −2.0) (Fig. 3A eFigure 2), reflecting lower levels of physical health. In comparison to those without obesity, the new obesity group had higher odds of severe fatigue in the new obesity group (aOR: 2.1; 95% CI 1.4 to 3.2), while the persistent obesity group had no difference (aOR: 1.3; 95% CI 0.9 to 1.9; Fig 3B; eFigure 3). Compared to those without obesity, the new obesity and persistent obesity groups both had higher odds of severe dyspnea (aOR: 1.9 [95% CI 1.3 to 2.7] and aOR: 2.0 [95% CI 1.5 to 2.7], respectively). Compared to those without obesity, the PROMIS mental health score in the current LC cohort was worse in the new obesity group (−3.1 points; 95% CI −4.6 to −1.6) and in the persistent obesity group (−1.7 points; 95% CI −3.0 to −0.4), reflecting lower levels of mental health.

## Discussion

In this cross-sectional study of 3663 patients who were followed for over 3 years, we found that self-reported obesity was associated with worse long-term outcomes. Individuals with obesity were approximately twice as likely to have LC compared with individuals without obesity. In addition, those with obesity were more likely to experience worse outcomes across multiple validated, patient-reported outcomes measures including both physical and mental health-related functional outcomes. These findings provide important insights that can help drive better risk stratification and improve potential care pathways for these higher risk groups.

Our study demonstrated significantly lower PROMIS physical health scores in patients with obesity, as well as increased rates of severe fatigue and dyspnea. This aligns with prior studies showing impaired quality of life among individuals with obesity [28]. Notably, those authors found improvements in quality of life among those experiencing a ≥ 10% weight loss [28]. We also identified reduced PROMIS mental health scores, which aligns with data demonstrating the influence of obesity on mental health [28, 29].

To further explore the influence of obesity, we split our data into those with new obesity versus those with persistent obesity. We found there were no clinically meaningful differences between those with new versus persistent obesity across the physical and mental health outcomes. While many factors can contribute to the development of obesity, this adds to the existing literature on obesity in LC and provides evidence that obesity at any point is associated with LC and worsened quality of life.

The association between obesity and LC outcomes is likely multifactorial. Obesity has been demonstrated to impact immune responses mediated by both humoral and cellular mechanisms [14, 30]. While increased production of leptin and reduction in adiponectin can lead to direct immunologic stimulation, other mechanisms such as nutrient excess, adipocyte expansion, and local hypoxia may also trigger adverse cellular responses [14]. Studies have also identified higher rates of inflammatory mediators such as IL-6 in obesity, which has been proposed as a potential mediator of LC [31, 32].

The public health implications of these findings are substantial. Given the high prevalence of obesity and the number of individuals experiencing LC, many individuals may be at an elevated risk for long-term disability and reduced quality of life. This highlights a potential role for early identification, focused weight-reduction interventions, and ensuring sufficient resources are available for these populations. If confirmed with future research, health systems should consider integrating obesity management and health optimization into LC clinics and recovery pathways. The potential role of new aggressive weight reduction programs (e.g., with glucagon-like peptide-1 [GLP-1] medications) before the “next” infectious disease pandemic and the controlled study of GLP-1 medications as an LC intervention should be evaluated. The interplay between LC symptoms (e.g., fatigue, dyspnea) and reduced physical activity warrants further exploration, as LC symptoms contributing to lower exercise capacity and energy expenditure could predispose individuals to obesity over time [33, 34]. This potentially spiraling relationship underscores the importance of addressing exercise challenges and weight management holistically in LC recovery pathways.

The major limitation is that obesity was based on self-report. Although our obesity findings are consistent with population-level data [13], obesity is generally under-self-reported and even definitions of obesity may be in flux, making our findings hypothesis generating and need to be validated – or refuted – based on objective obesity data. Second, participant LC status was based on self-report, rather than objective testing or specific symptom criteria, and thus may include alternate conditions not reflective of LC. Recent research has highlighted challenges with definitions of LC across studies, with earlier definitions potentially having reduced sensitivity compared to self-report [35]. However, our approach is consistent with the most recent recommendations for defining LC, which emphasizes the myriad symptoms and importance of patient involvement with defining LC [10]. Third, our observational design was only able to demonstrate association, rather than causation. Fourth, it is possible that some participants may have developed obesity due to inactivity resulting from LC, rather than LC as a pathophysiologic factor. Fifth, we did not ask about use of newer weight-loss medications, although at the time of the pandemic these were not readily available. Sixth, obesity is closely linked to other comorbidities, such as diabetes, cardiovascular disease, and depression, which may independently influence LC outcomes. While we adjusted our models for multiple factors, we did not account for baseline physical or mental health, specific comorbidities, smoking status, or timing or severity of the initial SARS-CoV-2 infection. Therefore, future work should further explore the association between obesity and LC with a focus on potential contributing and amplifying factors. Finally, our population is predominately white, middle-aged, and located in the United States with high vaccination coverage and may not apply to other populations with different obesity or LC profiles.

## Conclusions

In this cross-sectional study of participants who were followed for up to 40 months after their initial SARS-CoV-2 index infection, we found that participants with self-reported obesity had nearly two-fold higher rates of self-reported current LC. Additionally, those with obesity had worse physical and mental quality of life compared to individuals without obesity. Future research should further evaluate the impact of obesity on LC.

## Supplementary Information

Supplementary Material 1.

## Data Availability

The datasets used and/or analyzed during the current study are available from the corresponding author on reasonable request.

## Abbreviation

CDC: Centers for Disease Control and Prevention
FSS: Fatigue Severity Scale
INSPIRE: Innovative Support for Patients with SARS-COV-2 Infections Registry
LC: Long COVID
MMRC: Modified Medical Research Council
PROMIS: Patient-Reported Outcomes Measurement Information System
STROBE: Strengthening the Reporting of Observational Studies in Epidemiology
